# Online Searching as a Practice for Evidence-Based Medicine in the Neonatal Intensive Care Unit, University of Malaya Medical Center, Malaysia: Cross-sectional Study

**DOI:** 10.2196/30687

**Published:** 2022-04-06

**Authors:** Nor Asiah Muhamad, Vinesha Selvarajah, Anuja Dharmaratne, Anushia Inthiran, Nor Soleha Mohd Dali, Nathorn Chaiyakunapruk, Nai Ming Lai

**Affiliations:** 1 Sector for Evidence-Based Healthcare National Institutes of Health Ministry of Health Shah Alam Malaysia; 2 School of Information Technology Monash University Malaysia Selangor Bandar Sunway Malaysia; 3 Department of Accounting and Information Systems University of Canterbury Christchurch New Zealand; 4 Institute for Medical Research National Institutes of Health Ministry of Health Shah Alam Malaysia; 5 Department of Pharmacotherapy College of Pharmacy University of Utah Salt Lake City, UT United States; 6 School of Medicine Taylor’s University Subang Jaya Malaysia

**Keywords:** evidence-based practice, online information searching, information retrieval, information seeking, clinical setting

## Abstract

**Background:**

The use of the internet for research is essential in the practice of evidence-based medicine. The online search habits of medical practitioners in clinical settings, particularly from direct observation, have received little attention.

**Objective:**

The goal of the research is to explore online searching for information as an evidence-based practice among medical practitioners.

**Methods:**

A cross-sectional study was conducted to evaluate the clinical teams’ use of evidence-based practice when making clinical decisions for their patients' care. Data were collected through online searches from 2015 to 2018. Participants were medical practitioners and medical students in a Malaysian public teaching hospital’s neonatal intensive care unit who performed online searches to find answers to clinical questions that arose during ward rounds.

**Results:**

In search sessions conducted by the participants, 311 queries were observed from 2015 to 2018. Most participants (34/47, 72%) were house officers and medical students. Most of the searches were conducted by house officers (51/99, 52%) and medical students (32/99, 32%). Most searches (70/99, 71%) were directed rather than self-initiated, and 90% (89/99) were completed individually rather than collaboratively. Participants entered an average of 4 terms in each query; three-quarters of the queries yielded relevant evidence, with two-thirds yielding more than one relevant source of evidence.

**Conclusions:**

Our findings suggest that junior doctors and medical students need more training in evidence-based medicine skills such as clinical question formulation and online search techniques for performing independent online searches effectively. However, because the findings were based on intermittent opportunistic observations in a specific clinical setting, they may not be generalizable.

## Introduction

### Online Searching Practice for Evidence-Based Medicine

Internet use for information searching has increased significantly since the early 21st century. There has been a significant increase in medical information searching because of the availability of health information online [1-3]. While searching for health information online allows laypeople to learn more about their health, medical experts search for information to make informed clinical decisions for their patients [4,5]. Evidence-based medicine (EBM) refers to the process of seeking medical information to make informed clinical decisions. The definition of EBM is “the conscientious, explicit, and judicious use of current best available clinical evidence, with the integration of clinical expertise, to make clinical decisions for the care of patients” [6]. Because the human brain has limited capacity, EBM allows medical practitioners to make decisions based on validated and reliable evidence. This will improve the overall health care quality by ensuring consistency of care provided to patients through informed clinical decisions [7,8].

Many medical practitioners have reported difficulties encountered while performing an EBM search. Among them are a lack of resources, a lack of search skills and experience, a lack of role models practicing EBM, and a lack of time to practice EBM [9-12]. These are the challenges that online EBM practitioners in resource-limited countries face [9,10,12-15]. One reason for this is the unavailability of adequate resources. As a result, it is critical to comprehend how information is sought during EBM practice in resource-limited country hospitals. Such research is limited and still in its early stages [9]. According to a review of literature from resource-limited countries in this context, interviews and questionnaires are used to investigate the challenges faced by online EBM practitioners [13,16-20]. These research studies may not provide data on real challenges encountered during a live information retrieval process. Examining real and live challenges can provide insight into actual searching behavior in situations where challenges with query expression and results review may arise during the information-seeking process. Thus, there is a need to investigate medical practitioners’ true searching behavior during live clinical rounds so that recommendations can be made based on the findings from actual searching challenges that arise during EBM practice.

This study focuses on online EBM practice in a resource-limited country, specifically Malaysia, which meets the World Bank’s definition [21]. Early research studies on EBM practices in Malaysia revealed that many medical practitioners are aware of EBM and have used the Cochrane database, and 6.7% of those polled have used MEDLINE to conduct a literature search [13]. According to these findings, the overall uptake of performing online EBM is lower. Malaysia, as a Southeast Asia—Optimizing Reproductive and Child Health in Developing Countries (SEA-ORCHID) project participant, took part in a large intervention project that took place throughout the region [22]. The SEA-ORCHID project sought to investigate how evidence-based teaching and practices are carried out in the departments of obstetrics and gynecology. Despite such an intervention, a recent study found that challenges remain around the knowledge and skills required for conducting searches for relevant information during EBM practice [12,23].

### Related Works

According to research, the practice of EBM requires medical practitioners to integrate 3 important aspects during clinical decision making: (1) the medical practitioner’s clinical expertise, (2) the best available evidence from multiple resources, and (3) patient values and preferences [8]. The second criterion is closely related to online information-seeking behavior and EBM. It is not the same as searching for information, in general, to be able to use the best available evidence from multiple sources. This is because the practice of EBM is governed by a specific set of procedures. As a result, only EBM-trained medical practitioners and allied health experts are known to be able to practice EBM [24]. Improper EBM practice, especially when searching for evidence, may result in the retrieval of incorrect or inappropriate information, posing threats and risks to patients’ lives. Medical practitioners must search multiple resources for validated and reliable evidence to support their medical decisions [24]. When making clinical decisions, medical practitioners were initially encouraged to rely on facts derived from books and printed materials as their primary sources of offline health information [25]. In recent years, however, medical information has been deployed and searched through online resources via information and communication technology (ICT) [26-30]. This indicates a shift in the EBM practice from offline to online. By providing access to online medical information, ICT facilitated the practice of online EBM [31-34].

Furthermore, the emergence of the internet and the World Wide Web sparked the development of online medical search domains and medical databases. Medical search domains and databases are designed with built-in customized search features to assist the searcher in finding relevant medical information in the shortest amount of time. Examples of such online medical search domains are PubMed, UpToDate, and the British Medical Journal. Information seeking is also an important part of the learning process, which includes searching, obtaining, and using information for evidence [35]. According to the findings of research studies, medical practitioners who use specialized medical information retrieval systems find them useful. They specifically reduced the amount of time needed to search for information and made it easier to incorporate searching into their medical workflow processes [4,26,36-38]. Nonetheless, it is critical to ensure that evidence is searched appropriately using ICT to retrieve only validated and reliable medical information during EBM practice [4,38].

### Information Searching Process Model

**Figure 1:**
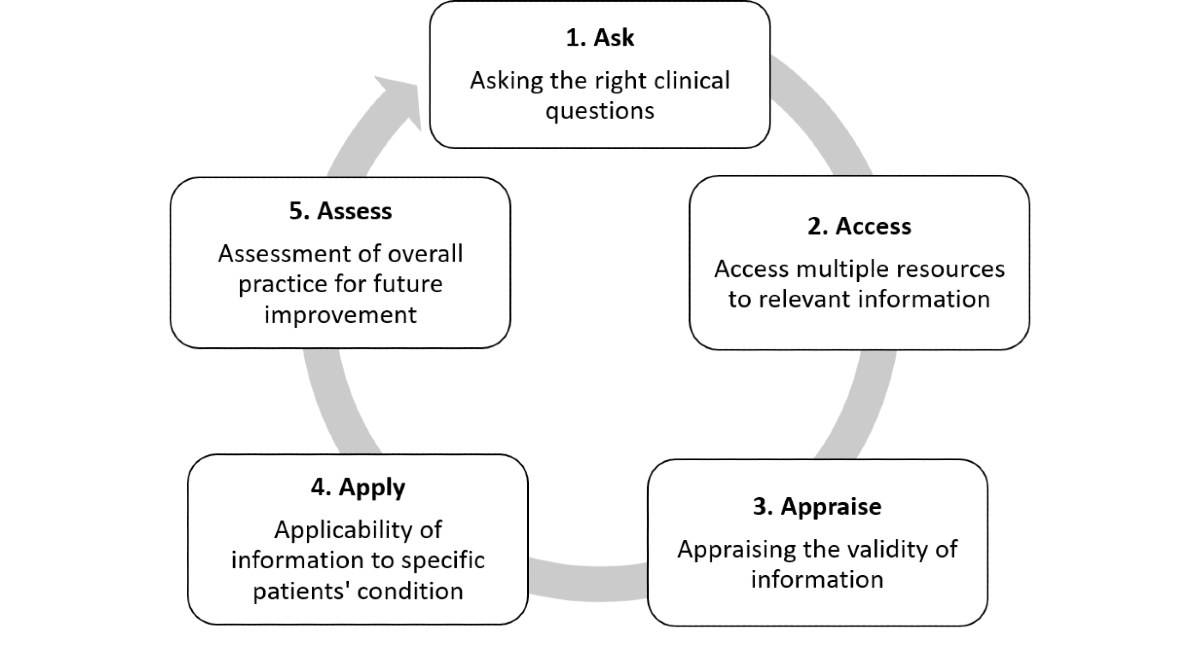
The 5-step guide to practice evidence-based medicine (adapted from Sackett [6]).

No evidence that explicitly defines the online searching process within the practice of EBM as the practice of EBM moved from offline searching to online searching. As a result, no search models exist to describe online EBM searching. However, EBM guidelines were developed to help medical practitioners practice EBM [24,39-41]. Sackett’s 5-step guide, depicted in Figure 1, is the most used guideline, and consists of 5 parts: inquire, access, appraise, apply, and evaluate [6].

**Figure 2:**
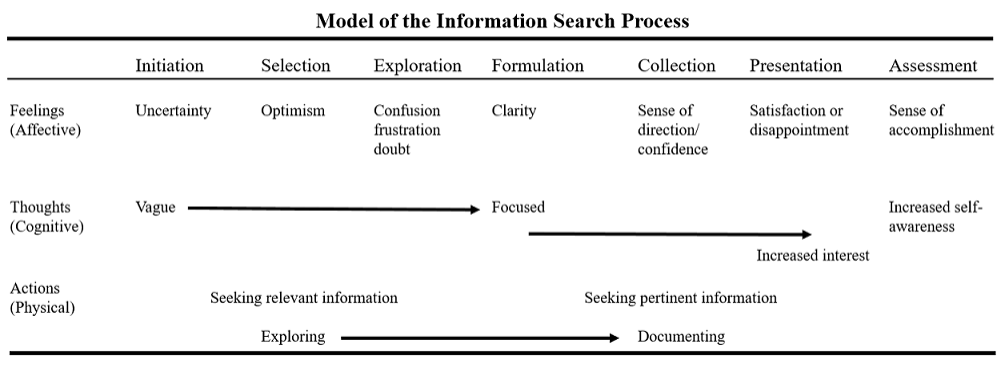
Information searching process model (adapted from Kuhlthau [42]).

As EBM practice has shifted to online, the information searching process (ISP) model (Figure 2) is the closest model that adapts to the online EBM searching process and describes the holistic experience of a typical searcher when searching for information [42]. It is the most appropriate model to explain the ISP within the context of EBM practice because it is a groundbreaking theory that models the holistic approach of a typical searcher. The ISP model is divided into 6 stages: initiation, selection, exploration, formulation, collection, and presentation [42]. All stages in the ISP model are adaptable and can be mapped to the EBM searching process.

When looking at the stages of the ISP model, there are 3 that are related to the online searching process: exploration, formulation, and collection. These stages describe the process of searching for information on the internet, including the formulation and reformulation of queries as well as the collection of desired information. They are analogous to the second step of the EBM guidelines, namely the access phase when practicing online EBM. In the EBM guidelines, the access phase denotes the process of searching for medical information (ie, accessing online resources to obtain information for clinical queries). As a result, the ISP model and EBM guidelines can be better classified as (1) querying behavior and (2) result viewing behavior. A thorough investigation of these behaviors was conducted in this study to provide a better understanding of online information-searching behavior during EBM practice. Another technique for searching for information, described by Bates [43], is insufficient because it involves interaction between the documents to be searched and the systems or browsers used, like the berry-picking process. The search for information will alter the overall search process, requiring users to investigate new information. This will provide the user with new directions to follow, which will change the search terms and queries.

### Previous Studies on Querying Behavior and Result Viewing Behavior

A few previous studies reported on querying behavior during the EBM practice [44-47]. These research studies were conducted on a single search domain and were based on self-perception of the search process. These studies did not include equally important aspects of querying behavior, such as the number of queries issued and query reformulation. According to the findings of research studies examining results viewing behavior during the practice of EBM, challenges are also encountered in this stage of the information seeking process [5,44,48,49]. Therefore, this study aims to explore and describe the online information-seeking behavior of EBM practitioners at the point of care where information-seeking activities were documented. Findings from this study can be used to design initiatives to improve the online searching process during the EBM practice.

## Methods

A cross-sectional study was conducted from 2015 to 2018 at the University of Malaya Medical Center, a tertiary teaching hospital in Malaysia.

### Setting

This study was conducted in the neonatal intensive care unit (NICU). The unit admitted 30 newborn infants per month on average, all of whom required critical care and constant monitoring. A clinical team of consultants, specialists, medical officers, science officers, nurses, and medical students will conduct clinical rounds at the NICU twice a day, every day (morning and evening). Two portable laptops were placed on a mobile trolley inside the NICU for use during clinical rounds. A few stationary desktop computers were also provided to aid in the search for clinical information. When a clinical question arose, members of the clinical team searched for answers using laptops or desktop computers preloaded with electronic databases such as the Cochrane Library and PubMed. The clinical team was told to search these electronic databases whenever they needed to for clinical queries.

### Ethics Approval

This study was approved by the Monash University Human Research Ethics Committee (project ID 4690). Further, the data collection methods for this research study were approved by the medical ethics committee of the University of Malaya Medical Council (MEC ID No. 201311-0506).

### Participants

A total of 47 participants were recruited from clinical teams that made clinical decisions for their patients’ care by constantly referring to the best empirical evidence. They were routinely involved in online search activities that took place during the study period between December 2015 and December 2018. A research assistant was present during these online activities.

### Data Collection

A standardized questionnaire containing information on the demographic characteristics of the participants was used to collect data. The questionnaire also included a structured observation involving the length of the search, time of the search, and method of search (either advanced or simple search) for 2 electronic databases. Before analysis, all data were deidentified. The process was documented through video recordings of the computer screen made with the Morae Manager (TechSmith Corp) key-logging recorder, and search terms were transcribed to a spreadsheet. Whenever a query was entered into the search field (eg, infan* or newborn or neonat* or premature or preterm or very low birth weight or low birth weight or VLBW or LBW) of the web browser or the search field of the search engine, the queries were observed and recorded using the Morae recordings. For both databases, a search strategy was developed. The keywords were identified before the search based on the clinical queries of participants/problems, interventions, comparisons, and outcomes (PICO). Using advanced search and Medical Subject Headings (MeSH) terms, similar terminology for each PICO will be identified. The literature search identified the information in all languages. The information collated included the number of queries, keywords used, length of the query (determined by calculating the number of terms/words used in a single query), use of Boolean operators (such as AND, OR) in queries, proportion of queries with typing and other errors, and the proportion of repeat queries to answer the same clinical question. For postsearch interviews, voice recordings were made and transcribed verbatim using NVivo (version 10, QSR International) software.

### Data Analysis

The data were analyzed for descriptive statistics using SPPS (version 22, IBM Corp) software for participant characteristics and online search, including querying pattern, resulting viewing pattern, and search duration. We presented the data collected from postsearch interviews narratively.

## Results

### Participant Characteristics

The characteristics of the participants are shown in Table 1. The participants included medical students, house officers, medical officers, and specialists.

**Table 1:** Demographic details of participants.

Variable			MS^a^ (n=15)		HO^b^ (n=19)		MO^c^ (n=8)		Specialist (n=5)
**Gender, n (%)**									
	Female	6 (40)		12 (63)		7 (88)		4 (80)	
	Male	9 (60)		7 (37)		1 (123)		1 (20)	
Age (years), mean (SD)			23.4 (0.6)		26.4 (1.9)		31.8 (4.1)		35.2 (5.7)
Age (years), range			23-24		24-32		26-39		28-41
**First language spoken, n (%)**									
	English	1 (5)		7 (40)		2 (25)		4 (80)	
	Malay	6 (40)		10 (50)		3 (38)		1 (20)	
	Chinese	7 (50)		2 (10)		2 (25)		0 (0)	
	Tamil	1 (5)		0 (0)		1 (13)		0 (0)	

^a^MS: medical student.

^b^HO: house officer.

^c^MO: medical officer.

### Reason for and Manner of Searches

Of the 99 searches, house officers conducted the most (51/99, 52%), followed by medical students (32/99, 32%), medical officers (10/99, 10%), and specialists (6/99, 6%). Most search activities were directed at junior members of the team, such as medical students and house officers, and self-initiated searches increased with seniority. Most of the search activities were carried out individually, with only a few carried out collaboratively (Table 2). Many participants used simple search strategies that consisted of one or more keywords entered alongside each other without the use of synonyms or any type of Boolean operator.

**Table 2:** The reason for and manner of searches.

Search type			MS^a^ (n=32), n (%)		HO^b^ (n=51), n (%)		MO^c^ (n=10), n (%)	Specialist (n=6), n (%)
**Search initiation**								
	Self-initiation	10 (31)		10 (20)		4 (40)		5 (83)
	Instructed	22 (69)		41 (80)		6 (60)		1 (17)
**Search activities**								
	Individual	30 (94)		46 (90)		8 (80)		5 (83)
	Collaborative	2 (6)		5 (10)		2 (20)		1 (17)

^a^MS: medical student.

^b^HO: house officer.

^c^MO: medical officer.

### Querying Activity

The querying activity represented the participants’ querying patterns during the search sessions. Whenever a query was entered into the search field of the web browser or search engine used to find information, the Morae recordings were observed and recorded. The querying activity’s results were presented in terms of participant categories and search types (foreground [FG] or background [BG]). FG refers to the user application and BG refers to the programs that are behind the scene. The results were further classified according to the number of queries issued, average query length, use of medical terms in queries, use of stop words and operators in queries, queries with spelling errors, issuance of ineffective queries, and reissuance of the same query. Table 3 shows the total number of queries issued and average query length.

**Table 3:** Number of queries issued and average query length of the queries issued.

Query		MS^a^ (n=15)		HO^b^ (n=19)		MO^c^ (n=8)			Specialist (n=5)	
		BG^d^ (s=26)	FG^e^ (s=6)	BG (s=45)	FG (s=6)	BG (s=8)	FG (s=2)	BG (s=3)		FG (s=3)
**Queries issued**										
	Sum	54	11	160	30	28	7	9		12
	Mean (SD)	2.08 (1.1)	1.83 (1.17)	3.56 (3.2)	5 (2.8)	3.5 (2.3)	3.5 (2.1)	3 (2)		4 (4.4)
	Range	1-5	1-4	1-14	2-9	1-7	2-5	1-5		1-9
**Query length, average**										
	Sum	98.6	42	159.1	23.4	28.7	9	18.2		14.6
	Mean (SD)	3.8 (1)	7 (2.6)	3.5 (1.4)	3.9 (1)	3.6 (2.3)	4.5 (2.1)	6 (1)		4.9 (4.4)
	Range	2-6	5-12	1.5-8	2.6-5	1.7-6	3-6	5-7		1-9.6

^a^MS: medical student.

^b^HO: house officer.

^c^MO: medical officer.

^d^BG: background.

^e^FG: foreground.

### Use of Medical Terms

Cross-checking the terms with medical terms in the MeSH library revealed the number of medical terms issued within a query. According to the results, 70.1% (218/311) of the queries issued were medical queries that included some medical terms. The participants used 307 medical terms, with an average of 1.4 medical terms per medical query recorded. The results also revealed that participants who frequently used medical terms in the queries were the house officers, who used an average use of 3.7 medical terms in the queries issued, as opposed to the medical students, medical officers, and specialists, who used a mean number of 2.3, 3.5, and 2.3 medical terms, respectively. The evidence from the participants’ verbal utterances suggested that they had included medical terms in their queries to retrieve more relevant results. Table 4 contains information on the medical terms used in the participant queries.

**Table 4:** Details of the use of medical terms in the queries issued.

Variable			MS^a^ (n=15)				HO^b^ (n=19)			MO^c^ (n=8)					Specialist (n=5)		
			BG^d^ (s=26)		FG^e^ (s=6)		BG (s=45)	FG (s=6)		BG (s=8)		FG (s=2)		BG (s=3)			FG (s=3)
**Medical terms**																	
	Sum	59		7		166		28	28		4		7			8	
	Mean (SD)	2.3 (2.2)		1.2 (0.9)		3.7 (4.2)		4.7 (4.9)	3.5 (4.2)		2 (1.4)		2.3 (3.2)			2.7 (3.8)	
	Range	0-8		0-3		0-16		0-13	0-12		1-3		0-6			0-7	
**Queries with medical terms**																	
	Sum	35		5		125		19	18		3		5			8	
	Mean (SD)	1.4 (1.1)		0.8 (0.4)		2.8 (2.8)		3.2 (2.8)	2.3 (2.1)		1.5 (0.7)		1.7 (2.1)			2.7 (3.8)	
	Range	0-4		0-1		0-11		0-7	0-6		1-2		0-4			0-7	
**Queries with medical terms, n (%)**																	
	Yes	29 (73)		5 (83)		39 (87)		4 (67)	7 (88)		—^f^		2 (67)			2 (67)	
	No	7 (27)		1 (17)		6 (13)		2 (33)	1 (13)		—		1 (33)			1 (33)	

^a^MS: medical student.

^b^HO: house officer.

^c^MO: medical officer.

^d^BG: background.

^e^FG: foreground.

^f^Not applicable.

### Use of Stop Words and Boolean Operators

According to the results of the analysis, stop words were used in only 65% (17/26) of the searches. The findings of this study differed from those of previous studies [45,46], which found that stop words were used in 80% of the searches conducted. The remaining searches lacked stop words in their queries, and the vast majority were BG-type searches. When examining the number of stop words used in the queries, FG-type searches had more stop words (1-4 stop words) than BG-type searches (1-3 stop words). The stop words used in this study were “in,” “of,” “on,” “is,” “for,” “and,” and “with.” The participants in this study did not frequently use Boolean operators in their queries (Multimedia Appendix 1). Only 4 of the searches had queries issued with Boolean operators, which were issued by the house officers. In this study, the operators used were the double quotation mark, bracket, and AND operator.

### Search Activities

A total of 311 queries during 99 search sessions were issued by the participants, with a mean of 3.14 (SD 2.6) queries. Participants who were house officers issued the most queries (51/99, 52%), followed by medical officers (10/99, 10%), medical students (32/99, 32%), and specialists (6/99, 6%). The participants spent an average time of 2.3 hours per day searching for medical information, with a single medical information search lasting 21 minutes on average. The average number of queries issued by all participants ranged between 2 and 4 queries. The mean number of queries issued in the BG- and FG-type search categories differed slightly, with FG-type searches recording 3.5 (SD 2.7) queries, slightly higher than BG-type searches, which recorded 3 (SD 2.6) queries.

In total, 307 distinct medical-related keywords were used in the searches. The length of the participant queries were then checked. The length of a query was calculated based on the number of terms/words used in a single query. The average query length in this study was 3.9 (SD 1.76) words. Specifically, the mean query length for FG-type searches was 5.2 (SD 2.6) words, which was higher than the mean query length of BG-type searches, 3.71 (SD 1.4) words. Query length averages issued by house officers and medical officers were comparable, at 3.6 and 3.7 terms, respectively. Multimedia Appendix 2 depicts the issuance of queries with spelling errors, ineffective queries, and the reissuance of the same query. A total of 4% (4/99) of searches used Boolean operators, with "AND" being the only one. Spelling errors were found in 6.8% (21/311) of the queries. Participants were aware of the errors made after the search and reran the searches with the correct spelling.

### Result Viewing Activity

The number of results and sublinks clicked, number of tabs used to view results, and control functions used in searches were used to analyze participant result viewing activity. When participants used the search engine to access a specific link or webpage after the queries were issued, the number of results clicked was displayed (Multimedia Appendix 3). According to the findings of this study, 377 results were clicked when looking for information. Of these, 302 came from BG-type searches and the rest from FG-type searches. The mean number of results clicked in this study was 3.81 (SD 3.11). According to the data, the mean number of results clicked in FG-type searches was 4.41 (SD 3.043), while the mean number of results clicked in BG-type searches was 3.68 (SD 3.13). This indicates that there was a higher level of result viewing activity when searching for FG-related information.

When participants proceeded to click on the links presented in the result clicked/webpages visited, the number of sublinks clicked was recorded. In this study, the mean number of sublinks clicked was 1.27 (SD 2.43), with the mean value being higher in FG-type searches (1.82, SD 3.067) compared to BG-type searches (1.16, SD 2.29). The participants’ verbal utterances revealed that they clicked on the sublinks during the FG-type searches because of progressive searching within a result/webpage to gain a better understanding of the subject matter being searched.

An interesting pattern in the use of multiple tabs during searching was discovered during the analysis of the result viewing activity (Multimedia Appendix 4), which depicts the number of tabs opened and control functions used by participants. Of the total searches observed, 65.6% of participants viewed their results in more than one tab. The mean number of tabs used was 3.15 (SD 2.86). The mean number of tabs in FG-type searches was 4.29 (SD 3.53) compared to 2.91 (SD 2.66) in BG-type searches. This indicates that more tabs were opened during the result viewing process in FG-type searches than in BG-type searches. Participants who used control functions indicated that they were successful in finding the information they were looking for.

The analysis of the result viewing activities of the participants revealed the use of control functions when viewing results or webpages. In the postsearch interview, participants who had used control functions in their searches were asked why they had done so. Their responses were: “to improve the searching process” and “to skim through important content only.” In their result viewing activity, only 4 BG searches by a medical officer and 3 by medical students used control functions. CTRL-F (in 2 searches) and CTRL+ were the control functions used in this study. The CTRL-F function was used to search the information on the results page for terms like “defin,” “1P,” “size,” “mm,” and “pda.” In the results presented, the CTRL+ function was used as a zoom-in function to improve the viewing of images and text.

## Discussion

### Principal Findings

Previous studies evaluating search practices in health care trainees and practitioners relied heavily on participant perceptions of previous searches [13,23,44-47,50-52]. There have been no studies that have assessed search activities in an acute clinical setting based on direct observation of real-time searches as far as we are aware. Despite limitations in the period of engagement due to restrictions related to the nature of the NICU and disruption of the study period, our observations yielded useful information. The majority of those who took part were house officers who had been ordered by their superiors to conduct searches, usually alone. There were 3 queries per search session on average, with 4 words used in each query. Relevant evidence appeared to be found in more than three-quarters of cases, and in roughly two-thirds of cases, there was more than one source of relevant evidence. Junior members of the clinical team who were tasked with conducting searches to answer multiple questions posed by senior team members in a short time may encounter difficulties in locating the best evidence. This included ownership of queries, content expertise, search techniques, and the absence of another person to provide input into the search process. Due to time constraints or a lack of clarity in terms of the questions posed, the challenges may have resulted in errors and ineffective searches that were not followed up on. Identifying the best evidence among multiple sources could also be difficult, although this study did not assess how the searchers dealt with this. It has been demonstrated that increasing the use of medical terms in queries increases the likelihood of retrieving the desired information [45,46]. If well chosen from a focused clinical question, the average query length of 4 words in this study was usually sufficient for an effective quick search to retrieve some relevant evidence, either in a repository of primary studies such as PubMed or in preappraised resources such as the Cochrane Library [53].

There have been no previous studies that have reported findings on the result clicking behavior among medical students in terms of result viewing activity. In this study, the medical students used search tabs more frequently when looking for FG-related information. In addition, when compared to house officers, medical officers, and specialists, medical students used the most tabs while searching. When viewing the results of the click, the control functions were also used. In terms of the number of sources accessed, medical students accessed the fewest in the BG-type searches. The medical students indicated PubMed and MEDLINE as their preferred sources of information based on the searches they conducted.

In this study, house officers demonstrated the most active search behaviors. When compared to medical students, medical officers, and specialists, they conducted the most overall searches (54.5% of the total searches recorded in this study). During searches, house officers had the most querying activity (the highest average number of queries issued, the highest number of stop words used in queries issued for FG-type searches, the highest number of queries issued with spelling errors, and the most ineffective queries). The findings of this study contradict previous findings, which indicated a lower number of queries issued when participants searched for EBM-related information [44,45,47].

The medical officers’ search behaviors in this study were straightforward. They had completed 10.1% of all recorded searches, with 80% yielding successful outcomes. In their querying activity, they demonstrated simple search behaviors by issuing a higher average number of queries and a longer average query length. They also used the fewest stop words in both their FG- and BG-type searches, had no spelling errors in their queries, and included medical terms in all of their FG-type searches. Although the simple search behaviors demonstrated by medical officers were effective in producing successful outcomes, the findings of this study do not agree with previous research findings. Previous research found that the number of queries issued was lower and the average query length was shorter [44,45,47].

In this study, the specialists displayed 2 types of searching behaviors: uncertain and expert. The specialists’ uncertain behavior was mirrored in their querying activity. Specialists issued the most queries in FG-type searches compared to BG-type searches, the longest average query length in BG-type searches, the fewest medical terms used in FG-type searches, and the most stop words used in all BG-type searches. In addition, specialists issued a greater number of ineffective queries in their BG-type searches than in their FG-type searches. Such behaviors by specialists were classified as uncertain and differed from previous studies, which reported fewer queries issued, a greater number of medical terms used, and the use of stop words to prevent the searcher from searching for their desired information [44-47,54]. This uncertain behavior of the specialists indicates the need for better information retrieval strategies to improve their online searching behaviors during the EBM practice. The findings point to a possible focus on training to improve the effectiveness of searches. These include the target participants of junior doctors and medical students, techniques for converting clinical encounter queries into well-constructed questions with relevant keywords, recognition of types of research that are most likely to answer specific questions, ranking of keywords to determine their order in searches, and appropriate use of Boolean operators.

### Limitations

Our study has some limitations. First, our findings may not be generalizable because they were conducted in a clinical practice setting with a specific team structure and facilities. Although most hospitals have a hierarchical structure like the one used in our study, the nature of task delegation, particularly in information retrieval, may differ across countries. Furthermore, because the search sessions recorded in this study were limited to selected morning clinical rounds, the information gathered over a very limited cumulative engagement period may not represent the participants’ true search behaviors. The type and amount of prior training received by the searchers may differ, which may result in different search patterns and results. On the other hand, as part of evidence-based practice, the NICU studied was provided with devices conducive to online searching and such facilities may not be widely available in places with limited resources.

### Conclusion

In conclusion, our study found that junior doctors were the primary individuals tasked with searching for clinical evidence in the NICU of a tertiary hospital in Malaysia. They mostly searched on their own, using simple, quick search strategies based on a few keywords. In three-quarters of the cases, they recovered what appeared to be relevant evidence, failing in one-quarter of the cases. This suggests that different search behavior profiles are required among the various types of EBM practitioners. The searches recorded in this study were based on clinical problems encountered by the EBM practitioners to reflect the participants’ true search behaviors. According to the findings of this study, different online searching behaviors were observed during the practice of EBM among different types of EBM practitioners. More research should be conducted on the facilitators and challenges of real-time searches in clinical settings, types of questions asked, and quality of evidence retrieved, as well as the association between effective evidence retrieval and the provision of best evidence in guiding care and improvement in patient-important outcomes.

## Appendix

Multimedia Appendix 1 The use of stop words and operators in the queries issued.

Multimedia Appendix 2 The issuance of queries with spelling errors and ineffective queries.

Multimedia Appendix 3 Details of the number of results clicked and the number of sublinks clicked during result viewing activity.

Multimedia Appendix 4 Details of the number of tabs opened and control functions used during result viewing activity.

## Abbreviations

BG: background
EBM: evidence-based medicine
ICT: information and communication technology
ISP: information searching process
FG: foreground
LBW: low birth weight
MeSH: Medical Subject Headings
NICU: neonatal intensive care unit
PICO: participant/problems, interventions, comparisons, outcomes
SEA-ORCHID: Southeast Asia—Optimizing Reproductive and Child Health in Developing Countries
VLBW: very low birth weight
