# Supplementary material for: Online Searching as a Practice for Evidence-Based Medicine in the Neonatal Intensive Care Unit, University of Malaya Medical Center, Malaysia: Cross-sectional Study
Source: JMIR Form Res. 2022 Apr 6;6(4):e30687. doi: 10.2196/30687 (PMC9021944; doi:10.2196/30687)
Supplement: Multimedia Appendix 1 [file formative_v6i4e30687_app1.docx]

Multimedia Appendix 1: The use of stop words and operators in the queries issued

| **Variables** | **Participants (Number of participants)** | **MSs**  **(n=15)** | | **HOs**  **(n=19)** | | **MOs**  **(n=8)** | | **Specialists**  **(n=5)** | |
| --- | --- | --- | --- | --- | --- | --- | --- | --- | --- |
|  | **Type of Search (Number of Searches)** | **BG**  **(s=26)** | **FG**  **(s=6)** | **BG**  **(s=45)** | **FG**  **(s=6)** | **BG**  **(s=8)** | **FG**  **(s=2)** | **BG**  **(s=3)** | **FG**  **(s=3)** |
| **Number of Stop Words Used** | **Min** | 0 | 0 | 0 | 1 | 0 | 0 | 2 | 0 |
|  | **Max** | 5 | 4 | 18 | 14 | 6 | 3 | 10 | 17 |
|  | **Sum** | 37 | 16 | 92 | 34 | 9 | 3 | 20 | 17 |
|  | **Mean** | 1.4 | 2.7 | 2 | 5.7 | 1.1 | 1.5 | 6.7 | 5.7 |
|  | **SD** | 1.5 | 1.6 | 3.2 | 5 | 2 | 2.1 | 4.2 | 9.8 |
| **Queries with Stop words** | **Yes** | 17  (65.4%) | 5  (83.3%) | 26  (57.8%) | Yes | 4 (50%) | 1  (50%) | Yes | 1  (33.3%) |
|  | **No** | 9  (34.6%) | 1  (16.7%) | 19  (42.2%) |  | 4 (50%) | 1  (50%) |  | 2  (66.7%) |
| **Queries with Operators** | **Yes** | No | | 2  (4.4%) | 2  (33.3%) | No | | | |
|  | **No** |  |  | 43  (95.6%) | 4  (66.7%) |  |  |  |  |
