# Supplementary material for: Online Searching as a Practice for Evidence-Based Medicine in the Neonatal Intensive Care Unit, University of Malaya Medical Center, Malaysia: Cross-sectional Study
Source: JMIR Form Res. 2022 Apr 6;6(4):e30687. doi: 10.2196/30687 (PMC9021944; doi:10.2196/30687)
Supplement: Multimedia Appendix 2 [file formative_v6i4e30687_app2.docx]

Multimedia Appendix 2: The issuance of queries with spelling error and ineffective queries

| **Variables** | **Participants (Number of participants)** | **MSs**  **(n=15)** | | **HOs**  **(n=19)** | | **MOs**  **(n=8)** | | **Specialists**  **(n=5)** | |
| --- | --- | --- | --- | --- | --- | --- | --- | --- | --- |
|  | **Type of Search (Number of Searches)** | **BG**  **(s=26)** | **FG**  **(s=6)** | **BG**  **(s=45)** | **FG**  **(s=6)** | **BG**  **(s=8)** | **FG**  **(s=2)** | **BG**  **(s=3)** | **FG**  **(s=3)** |
| **Number of Queries with Spelling Error** | **Yes** | 2  (7.7%) | No | 5  (11.1%) | 2  (33.3%) | 1  (12.5%) | No | 1  (33.3%) | No |
|  | **No** | 24 (92.3%) |  | 40 (88.9%) | 4  (66.7%) | 7  (87.5%) |  | 2  (66.7%) |  |
| **Number of Ineffective Queries** | **Yes** | 8  (30.8%) | 1  (16.7%) | 19 (42.2%) | 4  (66.7%) | 4  (50%) | Yes | 2  (66.7%) | 1  (33.3%) |
|  | **No** | 18 (69.2%) | 5  (83.3%) | 26 (57.8%) | 2  (33.3%) | 4  (50%) |  | 1  (33.3%) | 2  (66.7%) |
