# Supplementary material for: Online Searching as a Practice for Evidence-Based Medicine in the Neonatal Intensive Care Unit, University of Malaya Medical Center, Malaysia: Cross-sectional Study
Source: JMIR Form Res. 2022 Apr 6;6(4):e30687. doi: 10.2196/30687 (PMC9021944; doi:10.2196/30687)
Supplement: Multimedia Appendix 3 [file formative_v6i4e30687_app3.docx]

Multimedia Appendix 3: Details of the number of results clicked and the number of sub-links clicked during result viewing activity

| **Variables** | **Participants (Number of participants)** | **MSs**  **(n=15)** | | **HOs**  **(n=19)** | | **MOs**  **(n=8)** | | **Specialists**  **(n=5)** | |
| --- | --- | --- | --- | --- | --- | --- | --- | --- | --- |
|  | **Type of Search (Number of Searches)** | **BG**  **(s=26)** | **FG**  **(s=6)** | **BG**  **(s=45)** | **FG**  **(s=6)** | **BG**  **(s=8)** | **FG**  **(s=2)** | **BG**  **(s=3)** | **FG**  **(s=3)** |
| **Number of Results Clicked** | **Max** | 6 | 6 | 16 | 14 | 5 | 3 | 10 | 7 |
|  | **Min** | 1 | 2 | 0 | 5 | 1 | 1 | 2 | 1 |
|  | **Mean** | 2.69 | 3.83 | 4.4 | 6.5 | 2.38 | 2 | 5 | 3 |
|  | **SD** | 1.64 | 1.33 | 3.73 | 3.67 | 1.3 | 1.41 | 4.35 | 3.5 |
| **Number of Sub-Links Clicked** | **Max** | 6 | 7 | 13 | 3 | 4 | N/A | N/A | 11 |
|  | **Min** | 0 | 0 | 0 | 0 | 0 |  |  | 0 |
|  | **Mean** | 0.81 | 2.17 | 1.42 | 1.17 | 1.25 |  |  | 3.67 |
|  | **SD** | 1.47 | 2.79 | 2.77 | 1.47 | 1.75 |  |  | 6.35 |
