# Supplementary material for: Online Searching as a Practice for Evidence-Based Medicine in the Neonatal Intensive Care Unit, University of Malaya Medical Center, Malaysia: Cross-sectional Study
Source: JMIR Form Res. 2022 Apr 6;6(4):e30687. doi: 10.2196/30687 (PMC9021944; doi:10.2196/30687)
Supplement: Multimedia Appendix 4 [file formative_v6i4e30687_app4.docx]

Multimedia Appendix 4: The details of the number of tabs opened and control functions used during result viewing activity

| **Variables** | **Participants (Number of participants)** | **MSs**  **(n=15)** | | **HOs**  **(n=19)** | | **MOs**  **(n=8)** | | **Specialists**  **(n=5)** | |
| --- | --- | --- | --- | --- | --- | --- | --- | --- | --- |
|  | **Type of Search (Number of Searches)** | **BG**  **(s=26)** | **FG**  **(s=6)** | **BG**  **(s=45)** | **FG**  **(s=6)** | **BG**  **(s=8)** | **FG**  **(s=2)** | **BG**  **(s=3)** | **FG**  **(s=3)** |
| **Number of Tabs Opened** | **Max** | 6 | 9 | 17 | 15 | 4 | 2 | 12 | 5 |
|  | **Min** | 1 | 1 | 1 | 1 | 1 | 2 | 1 | 2 |
|  | **Mean** | 2.81 | 4.33 | 3 | 5.67 | 2 | 2 | 5 | 3 |
|  | **SD** | 1.55 | 2.81 | 3 | 5.05 | 1.2 | 0 | 6 | 1.73 |
| **Usage of Tab during search** | **Single Tab** | 7 (26.9%) | 1 (16.7%) | 22(48.9%) | 1 (16.7%) | 4 (50%) | No | 1(33.3%) | No |
|  | **Multiple Tabs** | 19 (73.1%) | 5 (83.3%) | 23 (51.1%) | 5 (83.3%) | 4 (50%) | Yes | 2(66.7%) | Yes |
